# Red Cross Balance Distributed

**Published:** 1921-03-05

**Authors:** 


### Red Cross Balance Distributed.

The Edinburgh Committee of the British Red Cross Society since last July has distributed £38,000 to the hospitals, being the balance in hand from the successful realisation of the assets in the hands of the Society after the Armistice had been declared. The value of these assets, which ran from materials to motor-cars, quickly increased, and they were sold, consequently, at a profit (so to speak) to the Society.

